# Long-term outcomes of drug-coated balloons in patients with diffuse coronary lesions

**DOI:** 10.3389/fcvm.2022.935263

**Published:** 2022-09-23

**Authors:** Xinyue Yang, Wenjie Lu, Liang Pan, Zhanying Han, Sancong Pan, Xi Wang, Yongjian Zhu, Yingguang Shan, Meng Peng, Peng Qin, Peisheng Zhang, Xiaofei Qin, Guoju Sun, Zhongsheng Qin, Jianzeng Dong, Chunguang Qiu

**Affiliations:** ^1^Department of Cardiovascular Medicine, The First Affiliated Hospital of Zhengzhou University, Zhengzhou, China; ^2^Department of Cardiovascular Medicine, Jincheng People’s Hospital, Jincheng, China; ^3^Department of Geriatric Cardiology, The First Affiliated Hospital of Zhengzhou University, Zhengzhou, China; ^4^Department of Cardiology, The Fifth Affiliated Hospital of Zhengzhou University, Zhengzhou, China

**Keywords:** coronary heart disease, percutaneous coronary intervention, diffuse coronary lesion, drug-coated balloon, drug-eluting stent

## Abstract

**Background:**

Drug-coated balloons (DCB), alone or in combination with drug-eluting stents (DES), may be used to treat diffuse coronary lesions. We aimed to explore the efficacy and safety of DCB in patients with diffuse coronary lesions.

**Methods:**

Consecutive patients with diffuse coronary lesions (lesion length > 25 mm) who underwent DCB and/or DES between January 2015 and December 2019 were included in this prospective, observational, multicenter study. The DCB group included 355 patients (360 lesions), of which 142 patients (143 lesions, 39.7%) received the DCB-only strategy and 213 patients (217 lesions, 60.3%) received the hybrid strategy (DCB combined with DES). The DES group included 672 patients (831 lesions) treated with DES alone. Target lesion revascularization (TLR) during 3-year follow-up was the primary outcome of interest. The secondary outcome was major adverse cardiac events (MACE), defined as a composite of all-cause death, non-fatal myocardial infarction, and target vessel revascularization.

**Results:**

The two groups had comparable baseline clinical and lesion characteristics. Lesion length was similar (43.52 ± 16.46 mm vs. 44.87 ± 15.80 mm, *P* = 0.181), but the stent length in the DCB group was significantly shorter (24.02 ± 23.62 mm vs. 51.89 ± 15.81 mm, *P* < 0.001). Ten lesions (2.8%) in the DCB group received bailout stents. Over 3 years of follow-up, no significant difference in TLR incidence between the groups (7.3 vs. 8.3%, log-rank *P* = 0.636) was observed. Incidence of MACE also did not differ significantly (11.3 vs. 13.7%, log-rank *P* = 0.324). No thrombosis events occurred in the DCB group, while four patients (0.6%) in the DES group experienced stent thrombosis (log-rank *P* = 0.193). Moreover, similar TLR and MACE rates were observed between DCB-only and hybrid strategies (TLR: 6.4 vs. 8.0%, log-rank *P* = 0.651; MACE: 11.4 vs. 11.2%, log-rank *P* = 0.884).

**Conclusion:**

Long-term outcomes show that the efficacy and safety of the DCB strategy (DCB alone or combined with DES) are similar to those of DES alone in diffuse coronary lesions. These findings suggest that this strategy is a promising alternative for select patients with diffuse coronary lesions.

## Introduction

Approximately 20% of the patients undergoing percutaneous coronary intervention (PCI) have diffuse coronary lesions (1). Patients with diffuse coronary lesions have greater cardiovascular risk factors and worse prognosis than those with non-diffuse lesions (2, 3). Despite advances in refined interventional technology and new-generation stents, PCI of diffuse coronary still remains problems such as stent thrombosis (ST) and vascular reendothelialization (4, 5). Given that stent length can separately predict in-stent restenosis (ISR) and thrombosis, using an alternative drug-coated balloon (DCB) to minimize excessive stent implantation has been recognized as a promising choice (2).

DCB were designed as a semi-compliant balloon that acts through surface anti-proliferative drugs. Multiple studies have shown that DCB alone is effective and safe in ISR (6, 7) and naive small vessel disease (8, 9). With increasing evidence, the Third Report of the International DCB Consensus Group (10) further updated the possible indications for DCB in patients with bifurcation lesions, large-vessel disease, and high bleeding risk. DCB alone or in combination with necessary “spot stenting” provides promising strategies to exploit the complementary advantages of DCB and drug-eluting stents (DES) in treating diffuse lesions. This method reduces the negative effects of permanent metal cages on native vessels (such as reendothelialization and side branch jailing) while also allowing for future revascularization. However, current clinical evidence is limited. Therefore, the goal of our research was to compare the long-term outcomes of DCB (DCB alone or combined with DES) and DES alone in treating diffuse coronary lesions.

## Materials and methods

### Population

**FIGURE 1:**
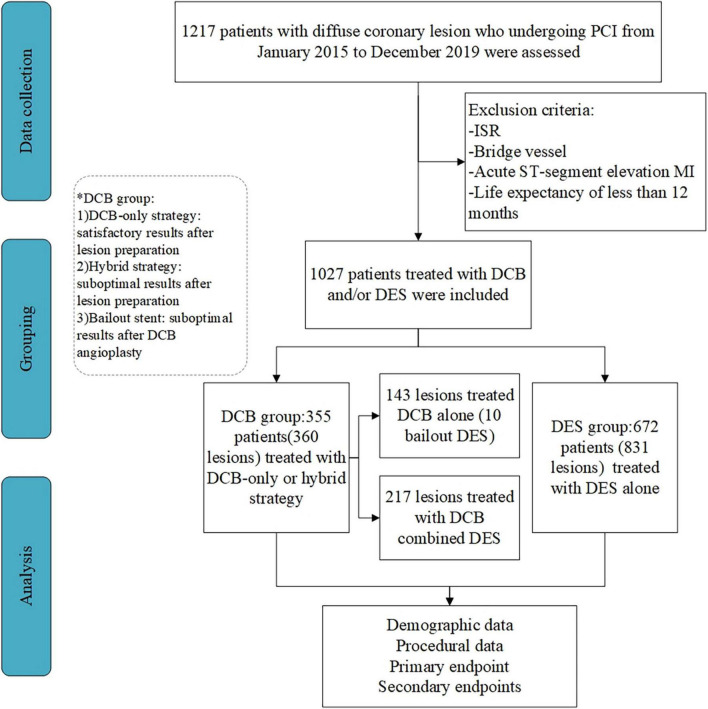
Flow of patient inclusion in the study. PCI, percutaneous coronary intervention; ISR, in-stent restenosis; CABG, coronary artery bypass grafting; MI, myocardial infarction; DCB, drug-coated balloon; DES, drug-eluting stent.

This prospective, non-randomized, observational study was conducted in three high-volume DCB centers in China (11–14). Consecutive patients with *de novo* diffuse coronary lesions (lesion length > 25 mm) who underwent DCB and/or DES implantation between January 2015 and December 2019 were included. Patients with ISR or bypass graft were excluded from the study. Additionally, we excluded patients with acute myocardial infarction (MI) or a life expectancy of less than 12 months. Figure 1 shows a flowchart of patient inclusion.

The local ethics committee approved the research and all patients signed written informed consent forms. This research was not sponsored by any external source.

### Procedures

PCI was performed following standard procedures. Patients were administered aspirin and a P2Y12 receptor inhibitor before surgery. Heparin was routinely administered during the procedure at a loading dose of 80–100 IU/kg followed by 1,000 IU per hour. Radial artery access was first considered, and the operators determined the use of glycoprotein IIb/IIIa receptor inhibitors. Dual antiplatelet therapy after discharge complied with international guidelines (15). The choice of interventional strategy (DCB or DES strategy) was left to the discretion of the experienced interventionist team after diagnostic coronary angiography.

In the DCB group, routine pre-dilatation with a conventional balloon was performed. To achieve satisfactory lumen gain, cutting balloons, non-compliant scoring balloons [non-slip element (NSE)], or dual-wire balloons were used at the discretion of the operators. According to lesion characteristics and pre-dilation results, the use of DCB alone or a hybrid approach of DCB and DES was determined by three experienced cardiologists. If pre-dilation is satisfactory, DCB was used to cover the entire target lesions or partial lesions in case of dissection < type C and residual stenosis ≤ 50%. New-generation DES implantation was performed as part of an initial hybrid strategy combining DCB and DES after lesion preparation or as bailout stents in segments with flow-limiting dissection or significant residual stenosis (>50%) after DCB angioplasty. All patients undergoing DCB angioplasty use paclitaxel−coated balloons (SeQuent™ Please, B. Braun, Melsungen, Germany) and new-generation DES was used in all patients as stents combined with DCB or bailout stents.

In the DES group, single, long stents or multiple overlapping stents were implanted to cover the entire lesion, and new-generation DES was used in all the patients. Four types of DES were implanted: Resolute Integrity™ (Medtronic, Santa Rosa, CA, USA), Synergy™ (Boston Scientific, Maple Grove, MN, USA), Excrossal (JW Medical System, China), Excel (JW Medical System, China).

### Angiographic analysis

Edge detection methods (QAngio XA 7.3 version; Medis Medical Imaging, Leiden, Netherlands) were used to provide quantitative coronary angiographic (QCA) measurements during the intervention and follow-up angiography. Lesion length, reference vessel diameter (RVD), minimum luminal diameter (MLD), and diameter stenosis percentage were measured. The difference in MLD immediately after intervention and during follow-up angiography was used to compute late lumen loss (LLL).

### Study outcomes

The primary outcome of this study was target lesion revascularization (TLR). The secondary outcomes were major adverse cardiac events (MACE) [including target vessel revascularization (TVR), MI, and all-cause death], respectively. During hospitalization, the major periprocedural adverse events included ST, MI, and death. Death was considered cardiogenic unless an apparent non-cardiogenic cause presented itself. The fourth universal definition of myocardial infarction is procedure-related, non-procedural, or after−discharge MI (16). Revascularization of the target lesion (a lesion within 5 mm from the edge of each end) and the target vessel was defined as TLR and TVR, respectively (17).

### Follow-up

Clinical visits or phone calls every 3 months were used to achieve follow-up for a total of 3 years. Follow-up angiograms were performed between 9 and 12 months after PCI or when clinically indicated, but this was not mandatory.

### Statistical analysis

The mean ± standard deviation or median (interquartile range) was used to represent data. The chi-square test or Fisher’s exact test was used to assess differences between categorical variables, and the *t*-test or non-parametric test was used for continuous variables. The Kaplan–Meier curve was used to estimate the cumulative incidence of outcomes, and the log-rank test was used to compare them. A multivariate Cox proportional regression model was used to adjust for potential confounders, including age, sex, comorbidities, history, multivessel disease, and left ventricular ejection fraction. Statistical analysis was conducted using SPSS 23.0 (IBM SPSS, SPSS Inc.). A two-sided *P* value of < 0.05 was considered statistically significant.

## Results

### Clinical and procedural characteristics

A total of 360 lesions in 355 patients received DCB alone or in combination with DES, and 831 lesions in 672 patients received DES alone, between January 2015 and December 2019. Baseline demographic and clinical data are shown in Table 1. The patients were aged 58.98 ± 10.93 years old and 68.6% were male. High rates of comorbidities were recorded, including hypertension (58.0%), diabetes mellitus (33.7%), and hypercholesterolemia (46.5%). Most patients had multivessel diseases (74.1%).

**TABLE 1:** Baseline patient characteristics.

	DCB group (*n* = 355)	DES group (*n* = 672)	*P*-value
Age, years	58.78 ± 11.12	59.08 ± 10.83	0.671
Male	246 (69.3)	459 (68.3)	0.778
**Comorbidities**			
Hypertension	209 (58.9)	387 (57.6)	0.740
Diabetes mellitus	113 (31.8)	233 (34.7)	0.368
Hypercholesterolemia	151 (42.5)	327 (48.7)	0.066
Previous MI	77 (21.7)	131 (19.5)	0.415
Previous PCI	49 (13.8)	84 (12.5)	0.559
Previous CABG	5 (1.4)	14 (2.1)	0.627
Previous stroke	54 (15.2)	120 (17.9)	0.295
Current/ex-smoker	151 (42.5)	293 (43.6)	0.791
Family history of CAD	94 (26.5)	143 (21.3)	0.062
Multivessel disease	276 (77.8)	485 (72.2)	0.061
LVEF, %	58.28 ± 8.03	58.68 ± 7.53	0.432

Values are mean ± SD or n (%). CABG, coronary artery bypass graft surgery; CAD, coronary artery disease; DCB, drug-coated balloon; DES, drug-eluting stent; LVEF, left ventricular ejection fraction; MI, myocardial infarction; PCI, percutaneous coronary intervention.

Most baseline features were similar between the DCB and DES groups. The trans-radial route was the main route of access (86.4% vs. 90.0%, *P* = 0.071). The DCB group had a larger proportion of chronic total occlusion (CTO) lesions (37.8 vs. 32.4%, *P* = 0.073). Cutting balloons (24.4 vs. 9.6%, *P* = 0.001) and NSE balloons (30.6 vs. 5.7%, *P* = 0.001) were more frequently used in the DCB group. Ten lesions (2.8%) in the DCB group required bailout stents (of which, seven were due to dissection ≥ type C and three were due to severe elastic recoil). The characteristics of the procedures and lesions are summarized in Table 2.

**TABLE 2:** Characteristics of procedures and lesions.

	DCB group (*n* = 360)	DES group (*n* = 831)	*P*-value
Access			0.071
Trans radial	311 (86.4)	748 (90.0)	
Trans femoral	49 (13.6)	83 (10.0)	
Target vessel			0.077
LAD	175 (48.6)	463 (55.7)	
LCX	81 (22.5)	164 (19.7)	
RCA	104 (28.9)	204 (24.6)	
Moderate/severe calcification	28 (7.8)	86 (10.4)	0.198
CTO	136 (37.8)	269 (32.4)	0.073
IVUS	125 (34.7)	198 (23.8)	< 0.001
**Pre-dilation**			
Semi-compliant balloon, %	341 (94.7)	824 (99.2)	< 0.001
Cutting balloon, %	88 (24.4)	80 (9.6)	< 0.001
NSE balloon, %	110 (30.6)	47 (5.7)	< 0.001
Dual wire balloon, %	5 (1.4)	11 (1.3)	> 0.999
**Dissection after DCB**			
Type A	32 (8.9)	/	
Type B	35 (9.7)	/	
Type C	6 (1.7)	/	
Type D-F	1 (0.3)	/	
TIMI flow grades after DCB/DES			0.438
Grade 3	357 (99.2)	829 (99.6)	
Grade 0–2	3 (0.8)	3 (0.4)	
Bailout stent	10 (2.8)	/	

Values are n (%). CTO, chronic total occlusion; DCB, drug-coated balloon; DES, drug-eluting stent; IVUS, intravascular ultrasound; LAD, left anterior descending artery; LCX, left circumflex artery; NSE, non-compliant scoring balloon; RCA, right coronary artery.

In the DCB group, 143 lesions (39.7%) received the DCB-only strategy, and 217 lesions (60.3%) received the hybrid strategy (DCB combined with DES). The mean length and diameter of DCB were 33.51 ± 16.60 and 2.69 ± 0.39 mm. DES implanted per lesion in the DCB group was significantly shorter (24.02 ± 23.626 vs. 51.89 ± 15.81 mm, *P* < 0.001) than in the DES group. Additionally, the average diameter of DES in the DCB group was smaller (2.84 ± 0.31 vs. 2.89 ± 0.39 mm, *P* = 0.047). There were no differences in perioperative medications (Table 3).

**TABLE 3:** Device characteristics and perioperative medication.

	DCB group	DES group	*P*-value
No. of patients/lesions	355/360	672/831	
**Treatment Strategy**			
DCB-only	143 (39.7)	/	
DCB combined with DES	217 (60.3)	/	
DCB in proximal of lesion	92 (25.6)	/	
DCB in distal of lesion	125 (34.7)	/	
**Device characteristics**			
DCB diameter, mm	2.69 ± 0.39	/	
DCB length, mm	33.51 ± 16.60	/	
DES diameter, mm	2.84 ± 0.31	2.89 ± 0.39	0.047
DES length, mm	24.02 ± 23.62	51.89 ± 15.81	< 0.001
**Perioperative medication**			
Aspirin	355 (100.0)	672 (100.0)	> 0.999
Clopidogrel	126 (35.5)	259 (38.5)	0.344
Ticagrelor	229 (64.5)	413 (61.5)	0.344
Glycoprotein IIb/IIIa inhibitor	235 (66.2)	405 (60.3)	0.068

Values are mean ± SD or n (%). DCB, drug-coated balloon; DES, drug-eluting stent.

### Quantitative coronary angiographic results

The lesion length was 43.52 ± 16.46 mm in the DCB group and 44.87 ± 15.80 mm in the DES group (*P* = 0.181). In the DCB group, the diameter of the reference vessel was 2.47 ± 0.45 mm, while in the DES group (2.88 ± 0.47 mm), it was larger (*P* < 0.001). Table 4 presents the QCA results. The MLD and diameter stenosis percentage before PCI did not differ significantly between the groups. However, MLD and acute gain immediately after PCI in the DCB group were smaller (1.79 ± 0.46 vs. 2.38 ± 0.54 mm, *P* < 0.001, and 1.31 ± 0.61 vs. 1.86 ± 0.65 mm, *P* < 0.001, respectively) than those in the DES group.

**TABLE 4:** Quantitative coronary angiography measurements.

	DCB group (*n* = 360)	DES group (*n* = 831)	*P*-value
**Before PCI**			
Lesion length, mm	43.52 ± 16.46	44.87 ± 15.80	0.181
RVD, mm	2.47 ± 0.45	2.88 ± 0.47	<0.001
MLD, mm	0.48 ± 0.48	0.52 ± 0.46	0.131
Diameter stenosis, %	80.78 ± 19.09	81.79 ± 15.55	0.379
**Immediately after PCI**			
MLD, mm	1.79 ± 0.46	2.38 ± 0.54	<0.001
Diameter stenosis, %	28.13 ± 11.00	17.98 ± 9.94	<0.001
Acute gain, mm	1.31 ± 0.61	1.86 ± 0.65	<0.001
Angiographic follow-up	157 (44.2)	293 (43.6)	0.895
RVD, mm	2.56 ± 0.51	2.77 ± 0.46	<0.001
MLD, mm	1.74 ± 0.59	1.94 ± 0.65	0.002
Diameter stenosis,%	31.96 ± 17.21	30.67 ± 18.80	0.622
Late lumen loss, mm	0.06 ± 0.61	0.41 ± 0.64	<0.001

Values are mean ± SD or n (%). DCB, drug-coated balloon; DES, drug-eluting stent; MLD, minimal luminal diameter; PCI, percutaneous coronary intervention; RVD, reference vessel diameter.

Follow-up angiography was performed in 157 patients (44.2%) who received DCB and 293 patients (43.6%) who received DES alone (*P* = 0.895). RVD (2.56 ± 0.51 mm vs. 2.77 ± 0.46, *P* < 0.001) and MLD (1.74 ± 0.59 vs. 1.94 ± 0.65 mm, *P* < 0.002) in the DCB group were smaller. However, the diameter stenosis was similar (31.96 ± 17.21% vs. 30.67 ± 18.80%, *P* = 0.622) between the groups. LLL in the DCB group was less than in the DES-only group (0.06 ± 0.61 vs. 0.41 ± 0.64 mm, *P* < 0.001) (Table 4).

### Study outcomes

**FIGURE 2:**
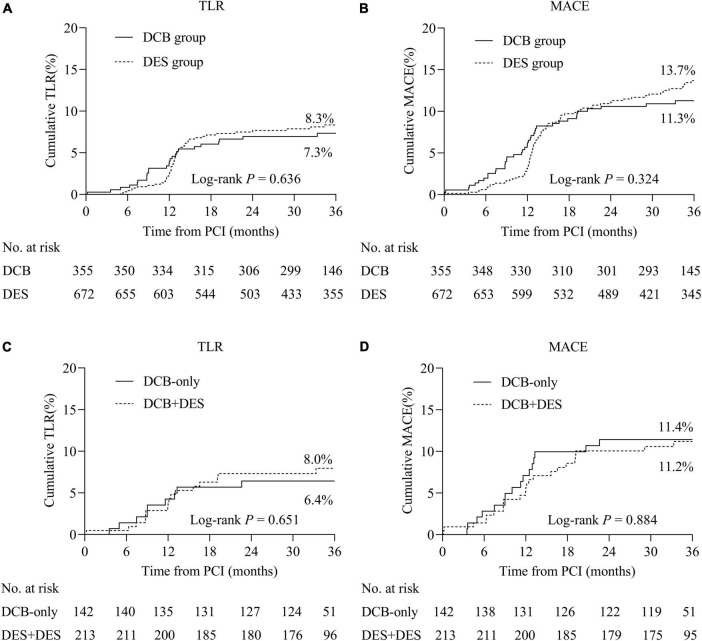
Cumulative incidence rates of primary and secondary outcomes. **(A)** TLR between the DCB and DES groups. **(B)** MACE between the DCB and DES groups. **(C)** TLR between the DCB-only and hybrid strategies. **(D)** MACE between the DCB-only and hybrid strategies. TLR, target lesion revascularization; MACE, major adverse cardiac events.

During hospitalization, one patient died in-hospital in the DCB group and another patient underwent periprocedural MI due to ST in the DES group (Table 5). The Kaplan–Meier survival curves of TLR and MACE between the DCB and DES groups are shown in Figures 2A,B. The primary outcome (TLR) rate at 3 years was 7.3% in the DCB group and 8.3% in the DES group (log-rank *P* = 0.636). The incidence of MACE at 3 years in the two groups was similar (11.3 vs. 13.7%, log-rank *P* = 0.324). The Kaplan–Meier survival curves for TLR and MACE between the DCB and DES groups are shown in Figures 2A,B. A multivariate Cox proportional regression model was used to adjust for potential confounders (Table 6). No difference in the risk of TLR was observed (HR = 1.229, 95% CI = 0.756–1.999, *P* = 0.405).

**TABLE 5:** Cumulative clinical events.

	DCB group (*n* = 355)	DES group (*n* = 672)	Log-rank *P*
**In-hospital events**			
MI	0	1 (0.1)	>0.999
ST (definite/probable)	0	1 (0.1)	>0.999
Death	1 (0.3)	0	0.346
**3-year follow-up**			
TLR	25 (7.0)	51 (7.6)	0.636
TVR	31 (8.7)	66 (9.8)	0.652
MI	2 (0.6)	9 (1.3)	0.282
ST (definite/probable)	0	4 (0.6)	0.193
All cause death	9 (2.5)	19 (2.8)	0.690
Cardiac death	6 (1.7)	14 (2.1)	0.820
MACE^†^	39 (11.0)	92 (13.7)	0.324

Values are n (%). ^†^MACE defined as the composite outcome of all-cause death, non-fatal myocardial infarction and target vessel revascularization (including periprocedural). DCB, drug-coated balloon; DES, drug-eluting stent; MACE, major adverse cardiovascular event; MI, myocardial infarction; ST, stent thrombosis; TLR, target lesion revascularization; TVR, target vessel revascularization.

**TABLE 6:** Multivariate cox regression analyses of TLR.

Variables	Hazard ratio (95% CI)	*P*
DES (vs. DCB)	1.229 (0.756–1.999)	0.405
Age (≥65 vs. <65)	0.686 (0.402–1.170)	0.167
Male	1.160 (0.666–2.019)	0.600
Hypertension	1.000 (0.628–1.592)	0.998
Diabetes mellitus	0.907 (0.556–1.482)	0.698
Hypercholesterolemia	0.902 (0.570–1.428)	0.660
Previous MI	1.273 (0.751–2.158)	0.371
Previous PCI	1.716 (0.963–3.059)	0.067
Previous CABG	1.084 (0.259–4.542)	0.912
Previous stroke	0.971 (0.521–1.810)	0.926
Current/ex-smoker	0.615 (0.368–1.029)	0.064
Family history of CAD	1.907 (1.177–3.091)	0.009
Multivessel disease	1.421 (0.808–2.497)	0.222
LVEF (≥50% vs. <50%)	0.786 (0.409–1.513)	0.472

The *P*-values for interaction are derived from Cox proportional hazard models. CABG, coronary artery bypass graft surgery; CAD, coronary artery disease; CI, confidence interval; DCB, drug-coated balloon; DES, drug-eluting stent; LVEF, left ventricular ejection fraction; MI, myocardial infarction; PCI, percutaneous coronary intervention; TLR, target lesion revascularization.

Within the DCB group, we performed analyses regarding the DCB-only and hybrid strategies (Figures 2C,D). TLR rates in patients treated with the DCB-only strategy were comparable to hybrid strategy (6.4 vs. 8.0%, log-rank *P* = 0.651). Between the DCB-only and hybrid strategies, the incidence of MACE was 11.4 vs. 11.2% (log-rank *P* = 0.884) (Supplementary Table 1). Regarding patients (*n* = 136) with CTO in the DCB group, 46 lesions (33.8%) received DCB-only strategy and 90 lesions (66.2%) received hybrid strategy. Patients with CTO treated with DCB-only or hybrid strategy showed comparable results (Supplementary Table 2).

## Discussion

This is the largest study to date to evaluate the long-term effects of DCB and DES in treating diffuse lesions. In this real-world trial, we discovered that in *de novo* diffuse coronary lesions, the long-term effectiveness and safety of PCI with DCB (DCB alone or combined with DES) were similar to those of DES alone.

Diffuse lesions, as a challenge of PCI, account for 20% of all coronary artery diseases (1). One of the most common procedures for treating diffuse lesions is PCI with a single-length DES or overlapping DES. On the other hand, long stent implantation increases the risk of ISR, ST, and late acquired stent malapposition (18). DCB was originally designed to treat ISR. However, increasing evidence has demonstrated the efficacy of DCB in small-vessel disease, acute MI, and bifurcation (10). A prior study (2) explored the efficacy of DCB in diffuse coronary lesions (length > 25 mm) and found that it was a reasonable and well-tolerated treatment option. It is worth mentioning that although sirolimus and its derivatives have recently been studied for treatment in DCB, PCI was performed only with paclitaxel-coated balloons in our study and we aimed to compare the long-term clinical outcomes of paclitaxel-coated balloons (DCB-only or hybrid strategy) with DES for diffuse lesions.

The TLR rate in the DCB group was similar to that in the DES group (7.3 vs. 8.3%, log-rank *P* = 0.636). Possible reasons for the lack of statistical difference in TLR include the following. First, the 3-year follow-up was not long enough. Second, new-generation DES and more potent antiplatelet therapy reduced the incidence of TLR and thrombotic events (19). Selective DCB angioplasty and experienced interventional physicians may improve the outcomes. Compared with a previous study (20) that evaluated the Zotarolimus-Eluting stents in diffuse coronary lesions (total stent length > 30 mm), the TLR rate of long-term (3 years) follow-up was numerically higher than that in our study (7.3 vs. 4.6%). Longer lesion length (43.52 ± 16.46 vs. 33.0 ± 15.2 mm) and smaller RVD (2.47 ± 0.45 vs. 2.73 ± 0.41 mm) in our study may explain the rate difference of TLR. Although our results showed that the outcomes in the DCB group were not superior to those in the DES group, the potential advantages of DCB, such as leaving nothing behind, lower thrombotic events, and late lumen enlargement, cannot be ignored.

In the current study, the metal stent length in the DCB group was significantly shortened per lesion compared with the DES group (24.02 ± 23.62 vs. 51.89 ± 15.81 mm, *P* < 0.001). Of the 360 lesions in the DCB group, 39.7% received DCB alone and the rest used a hybrid approach of DCB and DES. In contrast to previous studies with a combination of DCB mostly used in the distal segment and DES implanted in the proximal segment, the hybrid strategy of our study provides an idea that necessary spot stenting was performed in the segment with an unsatisfactory segment and that DCB angioplasty is more flexibly performed in any segment with acceptable results after lesion preparation. In the setting of diffuse lesions involving bifurcation or small vessel lesion, DCB provide an intervention option of “leave nothing behind” to avoid jailed ostial lesions or small caged vessel caused by DES. DCB without polymers and metal cages is beneficial for reducing the inflammatory response of coronary arteries, shortening the healing time of the vascular endothelium, and reducing the risk of thrombosis (10).

In our study, LLL was considerably lower in DCB group than in DES group (0.06 ± 0.61 vs. 0.41 ± 0.64 mm, *P* < 0.001) and QCA results demonstrated that a total of 46.3% of lesions appeared late lumen enlargement. The phenomenon of *de novo* lesions after DCB angioplasty has also been reported in other studies (21–26). This phenomenon has positive significance in particular lesions, such as CTO and small-vessel disease. Negative remodeling frequently occurs distal to the CTO lesion; therefore, the selected stent may be undersized, increasing the risk of ISR and limiting long-term revascularization (27). DCB has advantages over DES, permitting positive vessel remodeling without concern about stent malaposition. Within the patients (*n* = 136) with CTO in the DCB group, 46 lesions (33.8%) received DCB-only strategy and 90 lesions (66.2%) received hybrid strategy. Compared with previous studies (28, 29), DCB-only or combined with DES was effective and safe in treating selected CTO lesions during long-term follow-up. The acceptable results of subgroup indicate that diffuse coronary lesions after recanalization of CTO can be treated with DCB alone or combined with DES if the pre-dilatation result is favorable and provide more clinical evidence of DCB in treating CTO lesions.

Lesion preparation is of great significance for successful completion of the DCB strategy. The expert consensus (10) recommends that lesion preparation before DCB angioplasty should meet residual stenosis <30%, but there is insufficient evidence for the optimal lesion preparation criteria for treating *de novo* coronary lesions. A Korean study (30) analyzed more than 300 lesions and demonstrated that target lesion failure was lower with residual percentage diameter stenosis <20%. Theoretically, a lower residual diameter stenosis is better; however, excessive lesion preparation is accompanied by an increase in severe dissection, increasing the use of bailout stents. Ideal lesion preparation for DCB in the treatment of diffuse coronary lesions must balance the benefits and risks of acute lumen gain, plaque modification, acute vessel events, and long-term prognosis. In our study, considering that the plaque burden is heavier than simple lesions and avoiding the implantation of small-sized stents in distal lesions with small RVD, the standard for residual stenosis percentage before DCB angioplasty is defined as <50%. A total of 2.8% of lesions required a bailout stent in the DCB group, and one case required a bailout stent. Currently, DES is recommended as a bailout stent; however, it should still be avoided as much as possible.

Our study has some limitations. First, it was an observational study exiting selection bias despite a multivariate Cox proportional regression model was used to adjust for potential confounders. Second, angiographic follow-up is not mandatory, and angiographic follow-up varies. Third, since the choice of interventional strategy is based on clinical criteria and lesion types, operators may prefer DCB angioplasty for segments of target lesion with favorable pre-dilation results. Therefore, we cannot know the exact proportion of lesions that cannot be treated with DCB. Meanwhile, the proportion of IVUS use in the DCB group was higher than that in the DES group, which may influence clinical outcome. Our finding is only suitable for selected patients with acceptable results. Finally, our study didn’t directly compare the DCB-only or hybrid strategy with the DES-only strategy. Further larger, randomized, and controlled trials are necessary to separately evaluate the role of DCB-only or hybrid strategy in this setting.

In conclusion, research has shown that the use of DCB (DCB alone or combined with DES) in diffuse lesions is effective and safe. Therefore, this approach can be used as a supplement to DES or the preferred treatment in selected patients.

## Data availability statement

The original contributions presented in this study are included in the article/Supplementary material, further inquiries can be directed to the corresponding author/s.

## Ethics statement

The studies involving human participants were reviewed and approved by the First Affiliated Hospital of Zhengzhou University Jincheng People’s Hospital, Jincheng, the Fifth Affiliated Hospital of Zhengzhou University. The patients/participants provided their written informed consent to participate in this study.

## Author contributions

CQ, WL, JD, ZH, LP, and XY: conceptualization. XY, SP, XW, PQ, YS, YZ, PZ, and ZQ: data curation. CQ and XY: formal analysis. CQ, JD, and ZH: funding and acquisition. CQ, WL, JD, and ZH: methodology. CQ and ZH: project administration, resources, and supervision. CQ, ZH, GS, XQ, SP, and PZ: resources. XY: visualization. XY and WL: writing—original draft. All authors investigation, writing—review and editing, give final approval of the manuscript, and agreed to be accountable for all aspects of work ensuring integrity and accuracy.
