# Supplementary material for: Long-term outcomes of drug-coated balloons in patients with diffuse coronary lesions
Source: Front Cardiovasc Med. 2022 Sep 23;9:935263. doi: 10.3389/fcvm.2022.935263 (PMC9537625; doi:10.3389/fcvm.2022.935263)
Supplement: Supplementary file 1 [file Table_1.DOCX]

Supplementary Material

**TABLE 1 |** Cumulative Clinical Events.

|  | **DCB-only**  **(n = 142)** | **Hybrid strategy**  **(n = 213)** | **Log-rank *P*** |
| --- | --- | --- | --- |
| In-hospital events |  |  |  |
| MI | 0 | 0 | >0.999 |
| ST (definite/probable) | 0 | 0 | >0.999 |
| Death | 0 | 1 (0.5) | >0.999 |
| 3-year follow-up |  |  |  |
| TLR | 9 (6.8) | 16 (8.2) | 0.651 |
| DCB segment | 9 (6.8) | 5 (2.6) | / |
| DES segment | / | 7 (3.6) | / |
| Overlapping segment | / | 4 (2.0) | / |
| TVR | 14 (10.5) | 17 (8.7) | 0.544 |
| MI | 0 | 2 (1.0) | 0.245 |
| ST (definite/probable) | 0 | 0 | >0.999 |
| All cause death | 2 (1.5) | 7 (3.6) | 0.255 |
| Cardiac death | 1 (0.8) | 5 (2.6) | 0.229 |
| MACE^†^ | 16 (12.0) | 23 (11.7) | 0.884 |

Values are n (%). ^†^MACE defined as the composite outcome of all-cause death, non-fatal myocardial infarction and target vessel revascularization (including periprocedural). DCB, drug-coated balloon; DES, drug-eluting stent; MACE, major adverse cardiovascular event; MI, myocardial infarction; ST, stent thrombosis; TLR, target lesion revascularization; TVR, target vessel revascularization.

**TABLE 2 |** Cumulative Clinical Events of patients with CTO in the DCB group.

|  | **DCB-only** | **Hybrid strategy** | **Log-rank *P*** |
| --- | --- | --- | --- |
| No. of patients / lesions | 46/46 | 90/90 |  |
| In-hospital events |  |  |  |
| MI | 0 | 0 | >0.999 |
| ST (definite/probable) | 0 | 0 | >0.999 |
| Death | 0 | 0 | >0.999 |
| 3-year follow-up |  |  |  |
| TLR | 3 (6.5) | 9 (10.0) | 0.469 |
| TVR | 3 (6.5) | 10 (11.1) | 0.371 |
| MI | 0 | 2 (2.2) | 0.306 |
| ST (definite/probable) | 0 | 0 | >0.999 |
| All cause death | 0 | 4 (4.4) | 0.135 |
| Cardiac death | 0 | 4 (4.4) | 0.135 |
| MACE† | 3 (6.5) | 13 (14.4) | 0.172 |

Values are n (%). ^†^MACE defined as the composite outcome of all-cause death, non-fatal myocardial infarction and target vessel revascularization (including periprocedural). DCB, drug-coated balloon; DES, drug-eluting stent; MACE, major adverse cardiovascular event; MI, myocardial infarction; ST, stent thrombosis; TLR, target lesion revascularization; TVR, target vessel revascularization.
